# A novel cell factory for efficient production of ethanol from dairy waste

**DOI:** 10.1186/s13068-016-0448-7

**Published:** 2016-02-26

**Authors:** Jianming Liu, Shruti Harnal Dantoft, Anders Würtz, Peter Ruhdal Jensen, Christian Solem

**Affiliations:** National Food Institute, Technical University of Denmark, 2800 Kongens Lyngby, Denmark; Arla Foods Ingredients Group P/S, Sønderhøj 10-12, 8260 Viby J, Denmark

**Keywords:** *Lactococcus lactis*, Lactose catabolism, Residual whey permeate, Corn steep liquor hydrolysate

## Abstract

**Background:**

Sustainable and economically feasible ways to produce ethanol or other liquid fuels are becoming increasingly relevant due to the limited supply of fossil fuels and the environmental consequences associated with their consumption. Microbial production of fuel compounds has gained a lot of attention and focus has mostly been on developing bio-processes involving non-food plant biomass feedstocks. The high cost of the enzymes needed to degrade such feedstocks into its constituent sugars as well as problems due to various inhibitors generated in pretreatment are two challenges that have to be addressed if cost-effective processes are to be established. Various industries, especially within the food sector, often have waste streams rich in carbohydrates and/or other nutrients, and these could serve as alternative feedstocks for such bio-processes. The dairy industry is a good example, where large amounts of cheese whey or various processed forms thereof are generated. Because of their nutrient-rich nature, these substrates are particularly well suited as feedstocks for microbial production.

**Results:**

We have generated a *Lactococcus lactis* strain which produces ethanol as its sole fermentation product from the lactose contained in residual whey permeate (RWP), by introducing lactose catabolism into a *L. lactis* strain CS4435 (MG1363 Δ^3^*ldh*, Δ*pta*, Δ*adhE,* pCS4268), where the carbon flow has been directed toward ethanol instead of lactate. To achieve growth and ethanol production on RWP, we added corn steep liquor hydrolysate (CSLH) as the nitrogen source. The outcome was efficient ethanol production with a titer of 41 g/L and a yield of 70 % of the theoretical maximum using a fed-batch strategy. The combination of a low-cost medium from industrial waste streams and an efficient cell factory should make the developed process industrially interesting.

**Conclusions:**

A process for the production of ethanol using *L. lactis* and a cheap renewable feedstock was developed. The results demonstrate that it is possible to achieve sustainable bioconversion of waste products from the dairy industry (RWP) and corn milling industry (CSLH) to ethanol and the process developed shows great potential for commercial realization.

## Background

Currently, there is a growing demand for liquid fuels that can be produced in a sustainable manner from renewable raw materials. The potential that lies in using microorganisms for converting various feedstocks, e.g., plant biomass, into useful compounds including fuels, has already been recognized, and intense research is being carried out to establish robust and economically feasible processes for production of biofuels [1]. Despite its lower energy density and higher hygroscopicity compared to longer chain alcohols (butanol or pentanol) [2], microbially produced ethanol presently dominates the biofuel market, and it is mainly produced from either refined sugar or starch-derived sugar [3, 4]. Although much focus has been on developing bio-processes, which are reliant on non-food plant biomass as feedstock, there are many challenges, including the high cost of enzymes needed for degrading the biomass, the recalcitrance of lignocellulose, a lack of microbial catalysts with sufficient robustness to withstand the inhibitors generated in pretreatment or that have a sufficiently broad spectrum of carbohydrate utilization [5–8]. As an alternative, one cheap abundant feedstock is cheese whey and its various processed forms, such as whey permeate or whey powder. Whey is a liquid byproduct of cheese production, obtained when draining the cheese curd. The worldwide production of cheese whey in 2012 was reported to be 4 × 10^7^ tons and about 50 % hereof was used for animal feed or otherwise disposed of as waste [9]. The latter is a serious problem as whey is discarded as liquid waste and has a high BOD (biochemical oxygen demand) and COD (chemical oxygen demand) [9].

The composition of whey varies according to the source of the milk and the technology used for its production. Normally, it contains approximately 90 % water, 4 % lactose, 1 % protein, 0.7 % minerals, and small amounts of vitamins [10]. Separation of whey proteins generates whey permeate and further extraction of lactose leads to permeate mother liquor (residual whey permeate, RWP), as a leftover product [11]. Fermentation of the main carbon source in whey (lactose) to ethanol has been studied for the last 30 years and most of the research has been focused on yeasts that naturally metabolize lactose, such as *Kluyveromyces marxianus* or *Candida pseudotropicalis* [10–13]. Metabolic engineering of *Saccharomyces cerevisiae* for lactose fermentation has also been reported [14]. There are, however, problems associated with these microorganisms, and these include a general low robustness, slow fermentation rate, and substrate-inhibition effects, which is why there is a need for better performing microbial candidates [10–14].

*Lactococcus lactis*, which is well known for its role in cheese production, has been demonstrated to have great potential as a cell factory, due to properties such as its high glycolytic flux, ability to metabolize a broad range of carbohydrates, well-characterized metabolic network, and ease of genetic manipulation [15]. Its long record of safe use is also an important asset, especially for the production of food ingredients [16]. Normally, most of the carbon flux in *L. lactis* is directed to lactate (homo-lactic fermentation). However, it can be successfully engineered to produce ethanol by knocking out alternative product pathways and introducing pyruvate decarboxylase and alcohol dehydrogenase heterologously [17]. A potential drawback of using *L. lactis* as a cell factory is its fastidious nature, i.e., its many nutritional growth requirements, which could perhaps make it less attractive for some industrial applications, e.g., for production of low-priced chemicals where, for competitive reasons, it is important to keep costs at a minimum. However, relatively cheap fermentation media have been developed, and additionally, the availability of nutrient-rich waste substrates may help circumvent problems associated with using *L. lactis* as a production host organism.

**Fig. 1:**
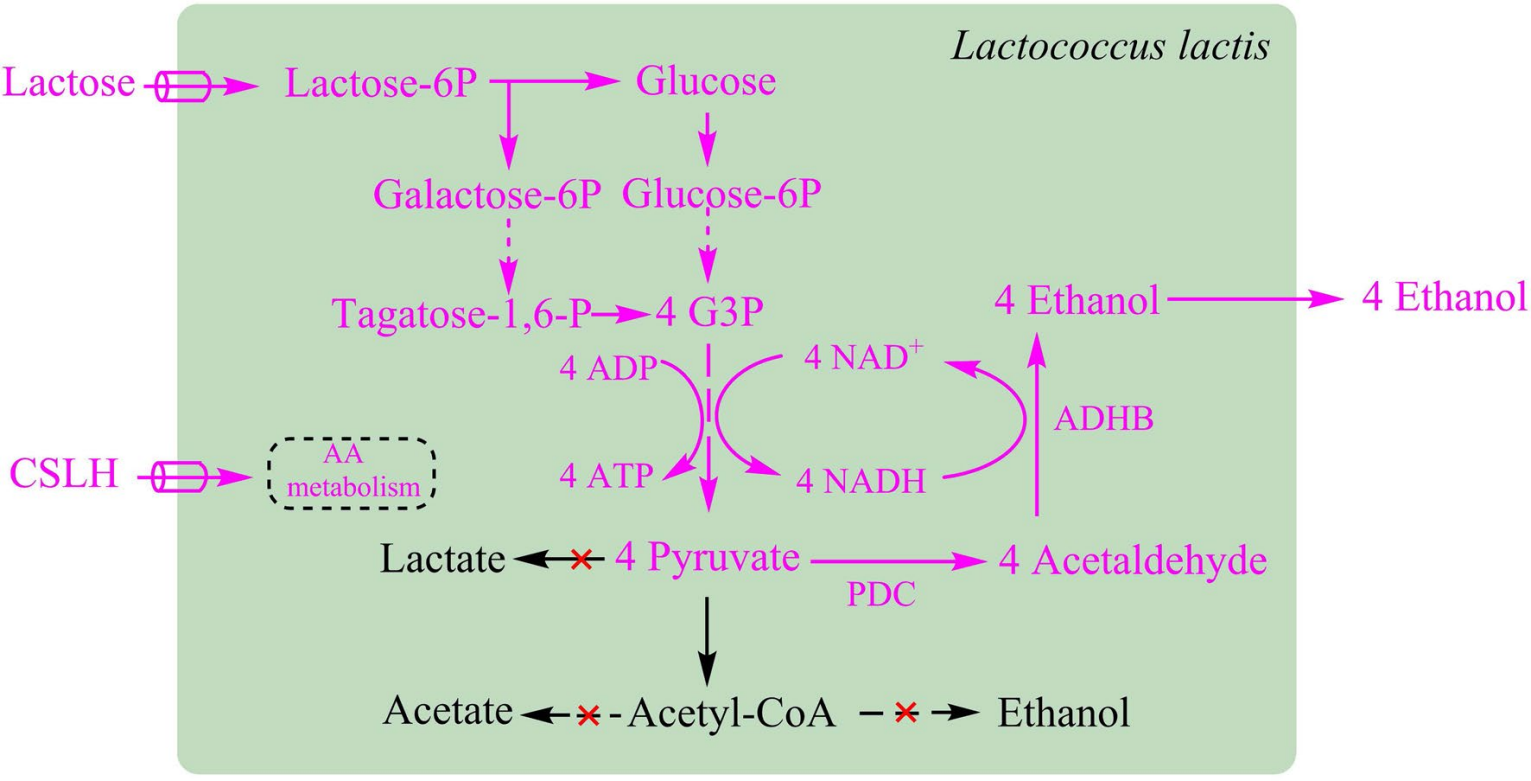
Efficient cell factory for ethanol production. The incorporation of heterologous ethanol-producing pathway enables complete cofactor recycling. Competitive pathways have been inactivated (indicated with *red mark*). *G3P* glyceraldehyde 3-phosphate, *PDC* pyruvate decarboxylase from *Zymomonas mobilis,* *ADHB* ethanol dehydrogenase from *Z. mobilis,* *AA* amino acids, *CSLH* corn steep liquor hydrolysate

In the current work, we have engineered *L. lactis* to produce ethanol by fermenting lactose and we demonstrate ethanol production in a medium based on a waste stream, residual whey permeate, from the dairy industry containing partially hydrolyzed Corn Steep Liquor (CSLH) as a nitrogen source (Fig. 1). By using a fed-batch strategy, we achieve high-level ethanol production with a titer of 41 g/L and a yield of 70 % of the theoretical maximum, which corresponds to 5.2 % (w/v) of ethanol in the broth. We not only demonstrate how a large industrial waste stream can be used for production of a useful value-added chemical, but also how an existing environmental problem can be alleviated.

## Results and discussion

### Redirection of *L. lactis* metabolism from homo-lactic to homo-ethanol

*L. lactis* is normally a homo-lactic fermentative bacterium where about 90 % of the glucose flux is directed to lactate (Table 1). As previously described [17], we successfully shifted the metabolic flux, of *L. lactis*, from lactate to ethanol by inactivating three lactate dehydrogenase (LDH) homologs −*ldh*, *ldhB*, *ldhX* (CS4099), the phosphotransacetylase (PTA, CS4234) and the native alcohol dehydrogenase (ADHE, CS4363) and introducing codon-optimized pyruvate decarboxylase (PDC)/alcohol dehydrogenase (ADHB) sourced from *Zymomonas mobilis*, and finally obtained the ethanol-producing strain CS4435. As shown in Table 1, acetate and ethanol became the dominant products after the deletion of LDHs, and the production of acetate could then be eliminated by deleting PTA. CS4363 was unable to grow under anaerobic conditions because of the defect in cofactor regeneration ability. However, its growth could be restored in the presence of O_2_, where NAD^+^ is recycled by NADH oxidase (NoxE) which results in acetoin as the main fermentation product. The introduction of PDC and ADHB restored complete cofactor recycling and bacterial growth under anaerobic conditions (Fig. 1), and the outcome was a homo-ethanol fermenting *L. lactis* (Table 1).

**Table 1:** Shifting the metabolism of *L. lactis* from homo-lactic to homo-ethanol

Strains	Description	Growth conditions	Carbon balance (%)	Lactose	Acetate	Formate	Acetoin	2,3-Butanediol	Ethanol
				mol/mol glucose					
MG1363	Wide type	Anaerobic	90	1.8 ± 0.1	ND	ND	ND	ND	ND
CS4099	MG1363 Δ^3^ *ldh,* (Δ*ldh*, Δ*ldhB* and Δ*ldhX*)	Anaerobic	85	ND	0.61 ± 0.02	1.58 ± 0.07	0.08 ± 0.01	0.09 ± 0.01	0.65 ± 0.02
CS4234	MG1363 Δ^3^ *ldh*, Δ*pta*	Anaerobic	88	ND	ND	0.82 ± 0.05	0.35 ± 0.02	0.16 ± 0.03	0.76 ± 0.04
CS4363	MG1363 Δ^3^ *ldh*, Δ*pta*, Δ*adhE*	Aerobic	85	ND	ND	ND	0.85 ± 0.05	ND	ND
CS4435	MG1363 Δ^3^ *ldh*, Δ*pta*, Δ*adhE* pCS4268	Anaerobic	87	ND	ND	ND	ND	ND	1.75 ± 0.06

Strains were cultivated in defined SA medium with glucose and samples were collected after 24 h. CO_2_ is included for calculating the carbon balance. Values are averages of three independent experiments and standard deviations are indicated

*ldh* (*ldhB*, *ldhX*) lactate dehydrogenase, *pta* phosphotransacetylase, *adhE* alcohol dehydrogenase, *pCS4268* pTD6-*pdc*-*adhB* (*pdc*, pyruvate decarboxylase from *Zymomonas mobilis,* *adhB* ethanol dehydrogenase from *Z. mobilis*)

### The incorporation of lactose catabolism in the ethanol-producing strain

**Fig. 2:**
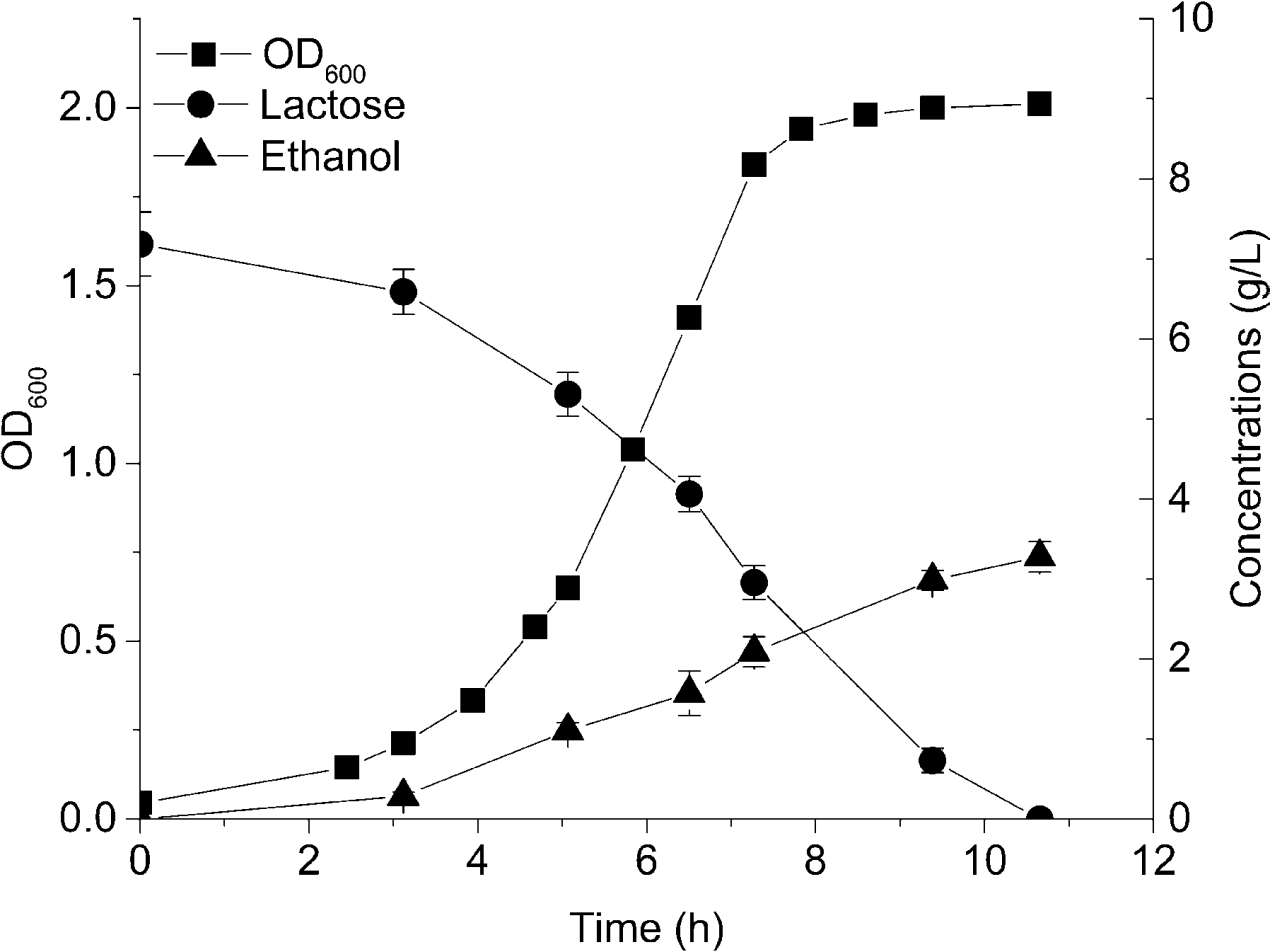
Characterization of CS4435L in defined medium containing lactose. CS4435L was grown in defined SA medium with 7.2 g/L lactose as the only energy source. Cell density (*filled squares*), lactose concentration (*filled circles*), and ethanol concentration (*filled triangles*) are displayed. Experiments were conducted in duplicate and *error bars* indicate standard deviations

CS4435 can produce ethanol as the sole fermentation product but cannot utilize lactose as a carbon source, since it is a derivative of the plasmid-free strain *L. lactis* MG1363 [18]. We therefore introduced the lactococcal plasmid-pLP712 (55.395 kbp), which encodes the entire lactose catabolism pathway [19], into strain CS4435 to make strain CS4435L, which could then grow on lactose. We then characterized the growth of CS4435L in defined synthetic amino acid (SA) medium [20] with 7.2 g/L lactose as the sole carbon source. As shown in Fig. 2, all the lactose were completely consumed within 11 h and the only fermentation product was ethanol. An ethanol concentration of 3.2 g/L was obtained with the yield of 0.45 g ethanol/g lactose, corresponding to 83 % of the theoretical maximum, with a growth rate close to 0.6 h^−1^. It was possible to increase the ethanol titer to 6.7, 10.3 ,and 12.0 g/L, when the initial lactose concentration was increased to 15.1, 24.0, and 31.8 g/L, respectively (Table 2).

**Table 2:** Optimization of lactose concentrations in different types of media

Lactose (g/L)	Media types	OD_600_	Ethanol (g/L)	Yield (g ethanol/g lactose)	Conversion efficiency^a^
7.2	SA^b^	2.0	3.2	0.45	0.83
15.1	SA^b^	2.3	6.7	0.44	0.82
24.0	SA^b^	2.9	10.3	0.43	0.80
31.8	SA^b^	3.6	12.0	0.38	0.71
32.0	RWP+YE^c^	7.2	13.4	0.42	0.78
40.0	RWP+CSLH^d^	4.0	17.5	0.44	0.82
80.0	RWP+CSLH^e^	6.0	30.6	0.40	0.71
80.0^g^	RWP+CSLH^f^	6.0	41.0	0.38	0.70

^a^Conversion efficiency was calculated based on the theoretical maximal yield (0.538 ethanol/g lactose)

^b^The composition of SA medium can be seen in reference 20. It includes 19 amino acids, vitamins, and salts. Glucose was replaced by lactose

^c^Diluted RWP (residual whey permeate) and 0.5 % (w/v) YE (yeast extract)

^d–f^Diluted RWP (residual whey permeate) and 2.5 % (w/v) CSLH (corn steep liquor hydrolysate), prepared by condition H1

^g^Fed-batch was performed with initial 80 g/L lactose and the details can be found in Fig. 7

**Fig. 3:**
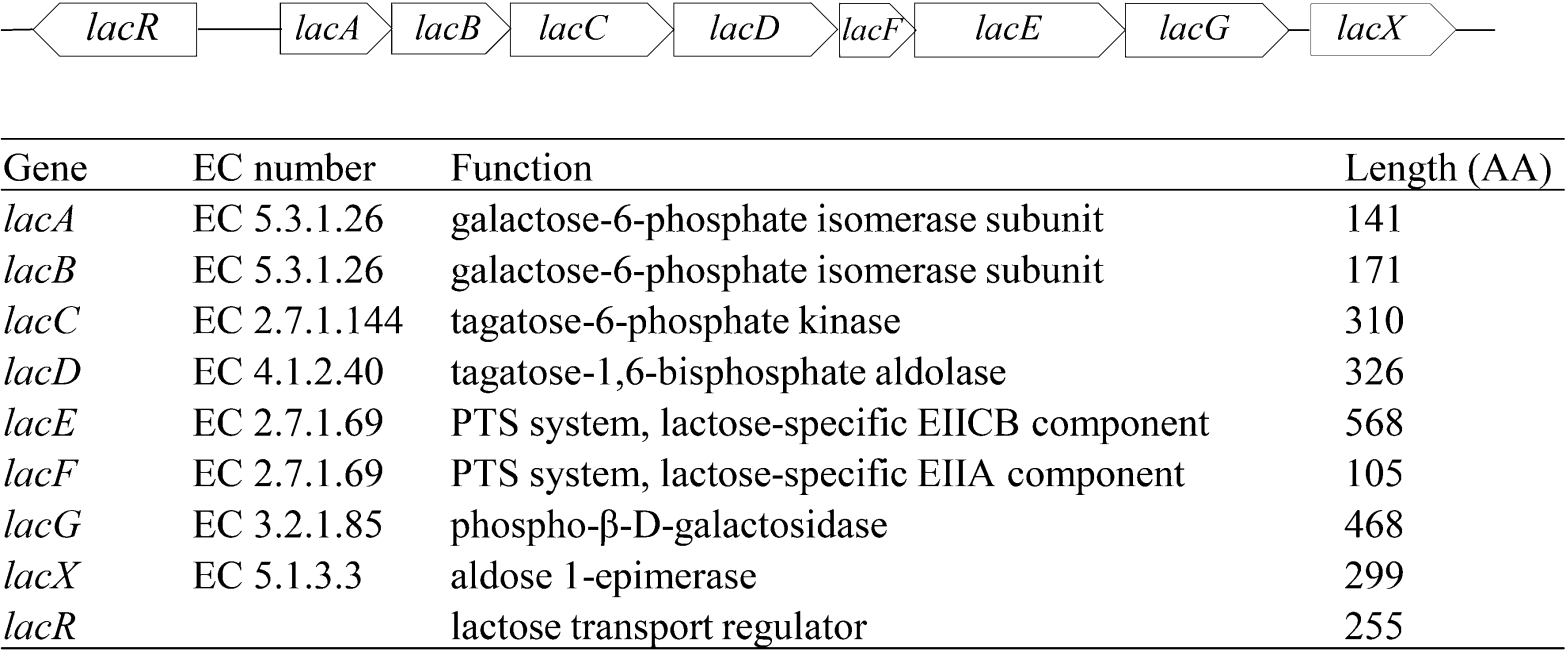
The lactose catabolism pathway. The location of each gene is displayed above and its EC number, specific function and amino acid (AA) length are summarized below

The results obtained clearly demonstrate the potential of *L. lactis* as a cell factory for converting lactose into value-added compounds. Dairy isolates of *L. lactis* are all able to metabolize lactose efficiently as they contain plasmids expressing the necessary genes. However, in our case, we started from a non-lactose-metabolizing laboratory strain, MG1363, which is a plasmid-free derivative of the dairy isolate NCDO712. MG1363 is easy to engineer, grows well, and has been well characterized, and this is the main reason for why it was chosen for the current study. The lactose plasmid which we introduced into the ethanol forming strain, pLP712, originated from NCDO712, which contains four other plasmids that potentially could interfere with genetic engineering. CS4435L is able to take up lactose via a lactose specific phosphotransferase system (PTS), encoded by *lacEF*, then phosphorylated lactose is hydrolyzed to glucose and galactose-6-phosphate (gal-6-P) by the phospho-β-galactosidase (*lacG*). The glucose moiety enters into glycolysis, while gal-6-P is degraded via the tagatose-6-P pathway (*lacABCD*). All the genes involved are located on the pLP712 plasmid (Fig. 3). Another way of metabolizing lactose has been reported, where lactose is transported by a lactose permease and cleaved intracellularly to glucose and galactose [21]. The galactose moiety is subsequently metabolized by the Leloir pathway. Since, in this case, lactose is taken up by a permease that depends on ATP hydrolysis, it is less efficient in terms of energy expenditure. For this reason, and because of its proven efficiency, we chose to use the PTS-based system. The growth rate obtained for CS4435L in lactose-SA medium was 0.6 h^−1^, which was only slightly below that obtained when growing on glucose (0.7 h^−1^) [20], which furthermore proved that the strategy chosen was the correct one.

### Development and optimization of a low-cost medium

**Fig. 4:**
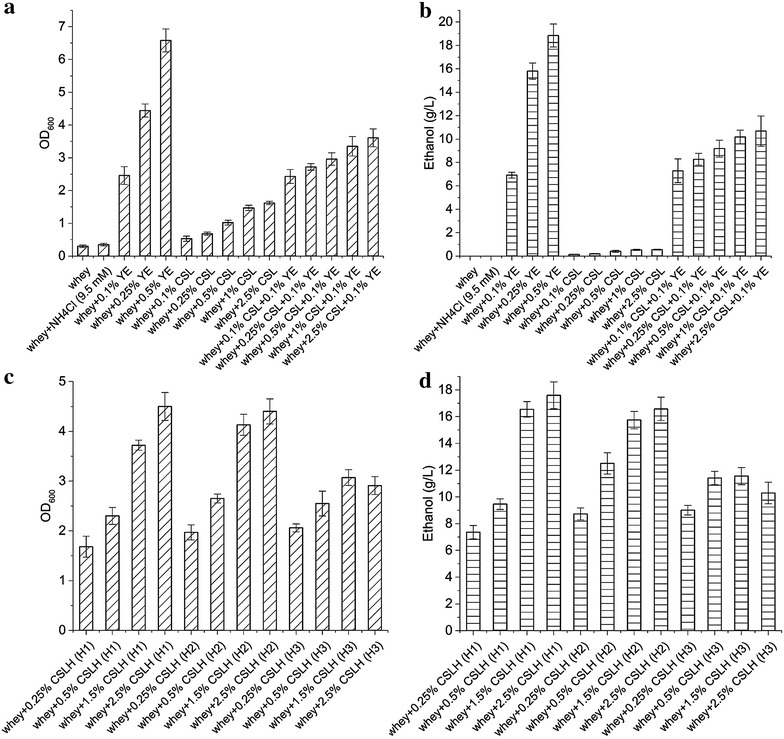
Optimization of low-cost medium for the ethanol forming strain CS4435L. Samples were collected after 30 h from cultures having initially 50 g/L lactose. **a**, **b** CSL, YE and their combinations;  **c**, **d** CSLH  only. The addition of YE, CSL, or CSLH is calculated as w/v. *YE* yeast extract, *CSL* corn steep liquor, *CSLH* corn steep liquor hydrolysate, *H1-H3* different hydrolysis conditions (H1: CSL treated with 0.05 % H_2_SO_4_; H2: CSL treated with 0.25 % H_2_SO_4_; H3: CSL treated with 0.5 % H_2_SO_4_). Experiments were conducted at least in duplicate and *error bars* indicate standard deviations

As mentioned above, CS4435L was able to grow well in a defined medium containing lactose as the sole energy source. In order to create a sustainable and economical bioprocess for ethanol production, we attempted to use a waste stream-residual whey permeate (RWP) as feedstock. As mentioned, RWP is the permeate mother liquor after extracting lactose from whey permeate. It was obtained from Arla Foods Ingredients Group P/S (http://www.arlafoodsingredients.com/) and its composition can be seen in Table 3. Relatively low amounts of amino acids were found to be present in RWP, and no cell growth or ethanol formation was observed in medium solely consisting of RWP (for all experiments three times diluted RWP containing 50 g/L lactose was used) (Fig. 4a, b). This was not surprising considering the amino acid requirements previously reported for *L. lactis* [20]. Therefore, different kinds of nitrogen sources at various concentrations were tested in order to optimize growth of the ethanol strain. Figure 4 shows that CS4435L was unable to grow in whey medium supplemented with inorganic NH_4_Cl, but the addition of yeast extract (YE) resulted in good growth. In RWP medium with 0.5 % (w/v) YE, a final cell density (OD_600_) of 6.5 was achieved after 12 h of fermentation, and the final ethanol concentration was nearly 19 g/L. According to previous studies, YE is the best choice, among various complex nitrogen sources, for supporting growth of lactic acid bacteria [22]. However, due to its high price (currently 7000 ~ 10,000 $/ton), it is not cost-effective to include it in a process for producing ethanol. In an economic analysis of microbial production of lactic acid, YE was estimated to contribute to over 30 % of the total production costs [23], which makes it necessary to find cheaper alternatives. Corn steep liquor (CSL), which is a byproduct of the corn milling industry, is a cheaper nitrogen source that currently costs around 500 $/ton. We tested CSL as a potential replacement for YE. At all concentrations [from 0.1 to 2.5 % (w/v)], however, the final biomass concentration was quite low with almost no ethanol produced. By combining 0.1 % (w/v) YE with CSL, a stimulation of growth was observed, but it was quite small (Fig. 4a, b). CSL did not support cell growth well and we speculated that it could be due to a low concentration of free and available amino acid concentration, as they could be locked up in insoluble proteins. To test this, we therefore attempted to hydrolyze CSL with different concentrations of H_2_SO_4_. These treatments involved very small amounts of sulfuric acid (0.05–0.5 % concentrated H_2_SO_4_ added to CSL with 20–30 % solid content). The H1 hydrolysate (CSL treated with 0.05 % H_2_SO_4_) provided the best results. When 2.5 % (w/v) corn steep liquor hydrolysate (CSLH) concentration was used, a high cell density of 4.5 (OD_600_) and a high ethanol titer (17.5 g/L) were obtained after 30 h of fermentation from 50 g/L lactose. A direct correlation between CSLH added and cell biomass, and ethanol production was observed (Fig. 4c, d). Increasing the amount of sulfuric acid in the pretreatment (H2, 0.25 % H_2_SO_4_) did not improve cell growth and ethanol production, and even had a negative effect at the highest concentration tested (H3, 0.5 % H_2_SO_4_). In the latter case, the cell density obtained was only 3.0 (OD_600_) and the final ethanol titer was 11 g/L when using 2.5 % (w/v) CSLH.

**Table 3:** The composition of residual whey permeate^a^

Composition	Concentration
Lactose	150 g/L
Galactose	3 g/L
Aspartate	0.252 mM (mmol/L)
Threonine	0.076 mM
Serine	0.088 mM
Glutamate	1.464 mM
Proline	0.384 mM
Glycine	0.904 mM
Alanine	0.24 mM
Cysteine	0.096 mM
Valine	0.072 mM
Methionine	0.124 mM
isoleucine	0.04 mM
Leucine	0.092 mM
Histidine	0.208 mM
Lysine	0.304 mM
Arginine	0.096 mM

^a^Residual whey permeate is a concentrate of the residue remaining after lactose extraction from whey permeate

CSLH proved to be a good source of nitrogen for *L. lactis* growth and efficient ethanol production, although the highest cell density obtained [4.5 (OD_600_) at 2.5 % (w/v) CSLH (H1)] was below that obtained in the presence of 0.5 % (w/v) YE (6.5). However, this should be considered as beneficial as biomass formation is often at the cost of ethanol yield. In our case, for *L. lactis*, the acid-digested CSL supported cell growth much better than untreated CSL, which may demonstrate that the protein present in CSL is unavailable for growth. Despite the fact that our strain possesses a cell-envelope bound protease encoded by genes present on plasmid pLP712, these proteins were apparently inaccessible, most likely because of their insoluble nature. This cell-envelope bound protease enables growth of *L. lactis* dairy isolates in milk, which is low in free amino acids and peptides [19]. In our case, the low level hydrolysis probably resulted in the formation of peptides and peptides are a preferred source of amino acids for this particular organism which possesses an impressive repertoire of intracellular peptidases as well as various uptake systems for peptides [24]. The negative effect of excessive hydrolysis (H3) may have caused a decrease in the amount of available peptides or perhaps could have resulted in formation of inhibitory compounds during acid hydrolysis [25, 26]. It is thus not likely that this simple pretreatment method would be beneficial for other microorganisms that are not adapted to growth in rich environments. Our work demonstrates that a fastidious microorganism should not be ruled out as a potential and perhaps superior industrial workhorse for the production of special chemicals [27].

### Ethanol production in the low-cost medium

**Fig. 5:**
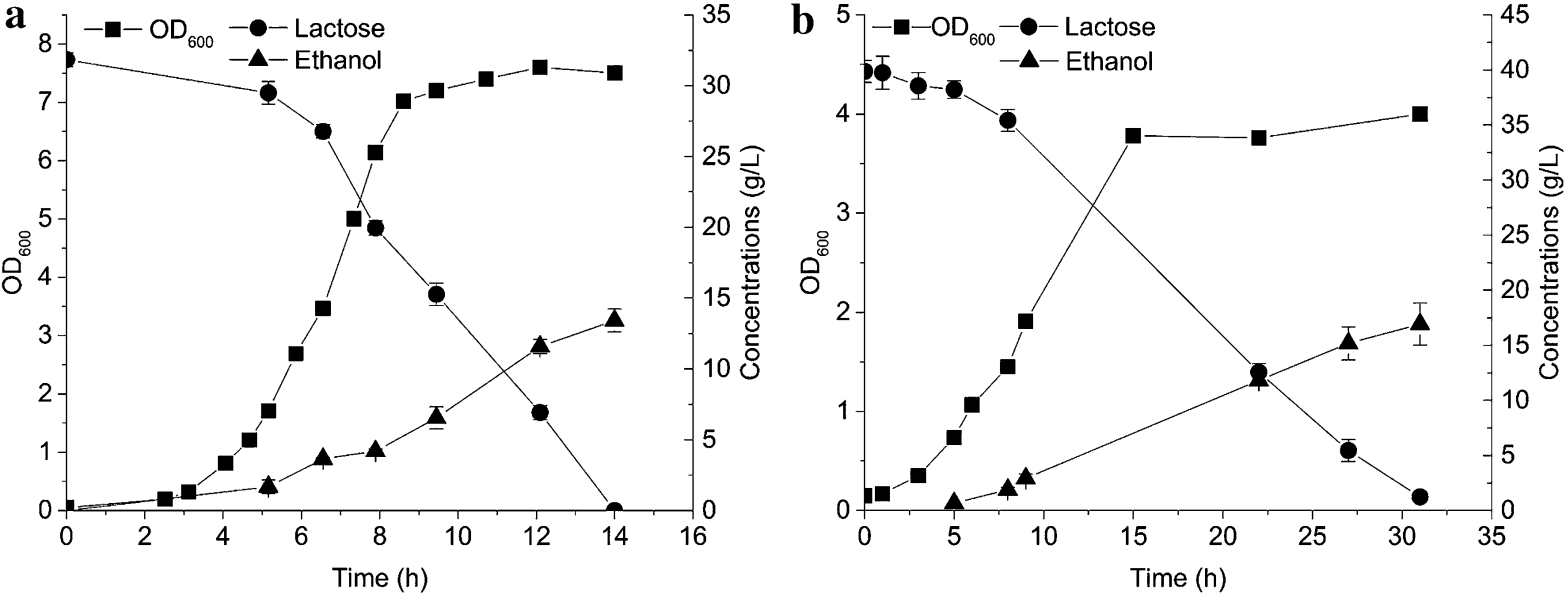
Comparing growth and ethanol formation of CS4435L in residual whey permeate medium with two different nitrogen sources. The medium only contains diluted residual whey permeate (RWP) without the addition of any vitamin or salt, except yeast extract (YE) or corn steep liquor hydrolysate (CSLH) as nitrogen sources. **a** The fermentation was carried out in diluted RWP medium containing initially 32 g/L lactose and 0.5 % (w/v) yeast extract; **b** The fermentation was carried out in diluted RWP medium containing initially 40 g/L lactose and 2.5 % (w/v) CSLH. CSLH was prepared based on H1 condition (CSL treated with 0.05 % H_2_SO_4_). Cell density (*filled squares*), lactose concentration (*filled circles*), and ethanol concentration (*filled triangles*) are displayed. Experiments were conducted in duplicate and *error bars* indicate standard deviations

For optimizing the growth medium, final cell density and ethanol titer were determined. To characterize growth as well as ethanol formation in more detail, fermentations were closely monitored. Medium containing YE was included for comparison. In Fig. 5b, it can be seen that 40 g/L lactose was completely consumed within 31 h and that the final cell density (OD_600_) reached 4.0 after 14.5 h in medium with 2.5 % (w/v) CSLH (H1). The ethanol concentration increased linearly to 17.5 g/L with 82 % of the theoretical maximum. However, in the presence of 0.5 % (w/v) YE, the ethanol yield was slightly less (78 % of the theoretical maximum), although growth was faster and the final cell biomass was higher (OD_600_ = 7.2, Fig. 5a). This can perhaps be explained in light of the fact that YE stimulates bacterial growth well and therefore more of the carbon flux is directed to cell biomass, leading to more biomass and less of the desired product. This is not ideal for production of chemicals and several studies have focused on reducing excess biomass formation to direct more of the carbon flux toward target chemicals. Becker *et al.* engineered *Corynebacterium glutamicum* to lower the biomass yield and finally got more lysine [28]. Hui Wu *et al*. obtained improved lactate concentration with lower biomass amounts [29]. In the present study, the replacement of YE with cheap CSLH resulted in a 44 % decrease in cell biomass yield but an overall increase in ethanol production yield (about 5 %).

The volumetric productivity of ethanol in the YE containing medium (0.96 g/L/h) was higher than that containing CSLH (0.56 g/L/h) (Fig. 5). We believe that the productivity in the CSLH medium can be improved through further medium optimization. It has been reported that the ethanol fermentation ability of *K. fragilis* was improved significantly when deproteinized whey (containing 0.5 % peptone) was supplemented with ergosterol and linoleic acid [30]. Adding ammonium sulfate or dipotassium hydrogen phosphate has also been demonstrated to lead to an improvement [31]. There is also the option of using adaptive evolution to improve performance in RWP-based media. On the other hand, the possibility to use YE for industrial ethanol fermentation cannot be excluded if the cost of YE becomes economically competitive. Finally, the option of combining YE with CSLH has not yet been tested, and could potentially improve both yield and productivity.

### Fed-batch fermentation

**Fig. 6:**
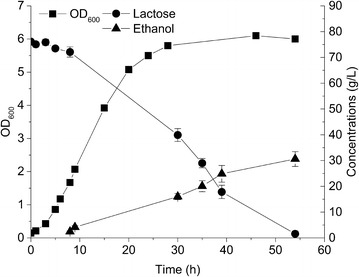
Time-course fermentation. Characterization of growth and ethanol production/lactose consumption in whey-based medium containing corn steep liquor hydrolysate (CSLH). The fermentation was performed with CS4435L in diluted residual whey permeate medium containing initially 80 g/L lactose and 2.5 % (w/v) CSLH. CSLH was prepared based on H1 condition (CSL treated with 0.05 % H_2_SO_4_). Cell density (*filled squares*), lactose concentration (*filled circles*), and ethanol concentration (*filled triangles*) are displayed. Experiments were conducted in duplicate and *error bars* indicate standard deviations

**Fig. 7:**
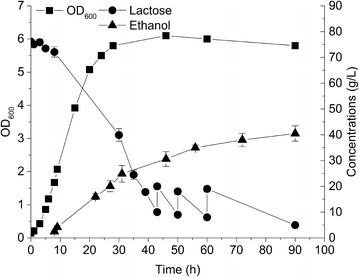
Fed-batch culturing of ethanol forming strain CS4435L for high-titer ethanol production. Fed-batch was performed with initial 80 g/L lactose and 2.5 % (w/v) CSLH in the residual whey permeate medium and 500 g/L lactose stock solution was used for feeding. CSLH was prepared based on H1 condition (CSL treated with 0.05 % H_2_SO_4_). Cell density (*filled squares*), lactose concentration (*filled circles*), and ethanol concentration (*filled triangles*) are displayed. Experiments were conducted in duplicate and *error bars* indicate standard deviations

Increasing ethanol titer in the fermentation broth is crucially important in order to reduce distillation costs in down-stream processing. It has been previously reported that at least 4 % (w/w) of ethanol should be present in the fermentation broth to lower the cost associated with distillation [32, 33]. We carried out fermentation experiments with an initial concentration of 80 g/L lactose, and the lactose was totally consumed in 55 h where the ethanol titer reached 30.6 g/L (yield of 71 %) (Fig. 6). We found that having more than 80 g/L lactose present from the beginning of the fermentation experiment was not beneficial in terms of ethanol titer, which may be due to a high osmotic pressure or decreased cell viability in the presence of higher ethanol concentrations [34, 35]. Because of this, a fed-batch fermentation strategy was devised and a final titer of 41 g/L ethanol was obtained after nearly 90 h with the yield of 70 % (Fig. 7). It was obvious that the ethanol formation rate was significantly lower in the last 40 h, demonstrating the weaker cell viability or lower enzyme activities as the fermentation time was extended [36].

These results are very encouraging and we believe that the titers and yields still can be improved. Koebmann *et al.* previously have demonstrated that the glycolytic flux in non-growing *L. lactis* can be increased after the introduction of F1-ATPase and similarly Hadicke *et al.* observed the lactate production rate increased in *Escherichia coli* when ATP consumption cycle incorporated [36, 37]. Both studies thus indicate that the ethanol flux can be further improved through energy manipulation inside the cells. Summing up, we have developed a robust strain that efficiently can convert a dairy waste product into a value-added product (ethanol) and we have demonstrated the potential of using the *L. lactis* chassis as a cell factory.

## Conclusion

We provide a good example of how *L. lactis* can serve as a cell factory, by engineering a derivative of *L. lactis* that can convert waste stream materials from the dairy industry into ethanol. The engineering efforts include substantial rewiring of metabolism, where we incorporate lactose metabolism into an ethanol producer (including the inactivation of LDH, PTA, ADHE, and introduction of heterologous PDC and ADHB). Finally, we achieve a high titer of 41 g/L ethanol with the yield of 70 % of the theoretical maximum by using a low-cost medium that contains a cheap nitrogen source (corn steep liquor hydrolysate) in combination with a fed-batch strategy. We believe that we may develop a cost-effective bioconversion process that can turn waste products from the dairy industry (RWP) and the corn milling industry (CSLH) into value-added ethanol which has immediate potential for commercialization.

## Methods

### Strains and plasmids

The plasmid-free strain *Lactococcus lactis* subsp. *cremoris* MG1363 or derivatives were used for the studies described in this article [18]. *Escherichia coli* strain ABLE-C (*E. coli* C lac (LacZ^−^)[Kan^r^ McrA^−^ McrCB^−^ McrF^−^ Mrr^−^ HsdR (r_k_^−^ m_k_^−^)][F´*proAB* lacI^q^ZΔM15 Tn10(Tet^r^)] (Stratagene) was used for cloning purposes. The lactose metabolism plasmid pLP712 (55,395 bp) was extracted from the dairy isolate NCDO712 based on the method of Andersen [38].

### Growth condition

*E. coli* strains were grown aerobically at 30 °C in Luria-Bertani broth [39]. For growth experiments, *L. lactis* was grown in 100 ml flasks without shaking in defined SA medium [20], where glucose was replaced by lactose, or residual whey permeate medium (RWP). RWP, which was provided from Arla Foods Ingredients Group P/S (http://www.arlafoodsingredients.com/), is the mother liquor from lactose production and its composition is shown in Table 3. When required, yeast extract (Sigma-Aldrich, USA) was used as a nitrogen source. Antibiotics were added in the following concentrations: erythromycin: 200 µg/ml for *E. coli* and 5 µg/ml for *L. lactis*, tetracycline: 8 µg/ml for *E. coli* and 5 µg/ml for *L. lactis*, chloramphenicol: 20 µg/ml for *E. coli* and 5 µg/ml for *L. lactis*.

### DNA techniques

All manipulations were performed according to Sambrook et al [39]. PfuX7 polymerase was used for PCR applications [40]. Chromosomal DNA from *L. lactis* was isolated using the method described for *E. coli* with the modification that cells were treated with 20 µg of lysozyme per ml for 2 h. Cells of *E. coli* were transformed using electroporation. *L. lactis* was made electro competent as described previously by Holo and Nes with the following modifications [41]: the cells were grown with 1 % glycine, and at an optical density of 0.5 (600 nm) ampicillin was added to a final concentration of 20 µg ml^−1^ and incubation was continued at 30 °C for 30 min.

### Analytical methods

Cell growth was regularly measured by OD_600_, and the quantification of lactose, glucose, lactate, formate, acetate, ethanol, acetoin and 2,3-butanediol was carried out using an Ultimate 3000 high-pressure liquid chromatography system (Dionex, Sunnyvale, USA) equipped with a Aminex HPX-87H column (Bio-Rad, Hercules, USA) and a Shodex RI-101 detector (Showa Denko K.K., Tokyo, Japan). For the detection of pyruvate, the DAD-3000 diode array detector (Dionex, Sunnyvale, USA) was used. The column oven temperature was set at 60 °C and the mobile phase consisted of 5 mM H_2_SO_4_, at a flow rate of 0.5 ml/min. As for the detection of amino acids in residual whey permeate, the filtered sample was first hydrolyzed with 6 M HCl and then separated by ion exchange chromatography and detected after oxidation and derivatization with *o*-phthaldialdehyde [42].

### Corn steep liquor hydrolysis

Corn steep liquor (CSL) was purchased from Sigma–Aldrich (St. Louis, MO) with 40–60 % solid content. Different hydrolysis conditions were applied to make corn steep liquor hydrolysate (CSLH). H1 condition: original CSL was diluted two times with water, and then 50 μl concentrated sulfuric acid was mixed with 100 ml diluted CSL. The mixture was kept at 121 °C for 15 min and subsequently pH was adjusted to 6.8–7.1 with the addition of 10 M NaOH solution. H2 condition: original CSL was diluted two times with water and then 250 μl concentrated sulfuric acid was mixed with 100 ml diluted CSL. The mixture was kept at 121 °C for 15 min and subsequently pH was adjusted to 6.8–7.1 with the addition of 10 M NaOH solution. H3 condition: original CSL was diluted two times with water and then 500 μl concentrated sulfuric acid was mixed with 100 ml diluted CSL. The mixture was kept at 121 °C for 15 min and subsequently pH was adjusted to 6.8–7.1 with the addition of 10 M NaOH solution.

### Ethanol fermentation

Defined SA medium was used for screening of lactose-metabolizing strain with lactose as the only carbon source. For nitrogen source optimization, 50 g/L lactose (diluted RWP) was mixed with different concentrations of nitrogen sources (NH_4_Cl, yeast extract, CSL or CSLH) in 25 ml tube with a volume of 10 ml. For ethanol production, RWP was diluted and used as the main substrate for fermentation without the addition of any vitamins or salts, except 2.5 % (w/v) CSLH. CS4435L was grown in a 125 ml flask with 100 ml of medium with slow magnetic stirring and no aeration. The cultivation was carried out at 30 °C. Fed-batch was performed with initial 80 g/L lactose and 2.5 % (w/v) CSLH, and 500 g/L lactose stock solution was used for feeding. The feeding was performed when the lactose concentration was lower than 10 g/L and after rapid injection it returned to around 20 g/L. Samples were collected periodically for determining cell density, lactose, and ethanol concentrations.

## Abbreviations

*L. lactis*: *Lactococcus lactis*
RWP: residual whey permeate
CSLH: corn steep liquor hydrolysate
YE: yeast extract
*ldh*: genes encoding for lactate dehydrogenase
*pta*: gene encoding for phosphotransacetylase
*adhE*: gene encoding for the native alcohol dehydrogenase
PDC: pyruvate decarboxylase from *Zymomonas mobilis*
ADHB: ethanol dehydrogenase from *Z. mobilis*. AA, amino acids
NADH or NAD^+^: reduced or oxidized form of nicotinamide adenine dinucleotide
OD_600_: optical density at wavelength of 600 nm
SA: synthetic amino acid medium
